# Redefining HIV-associated large-vessel vasculopathy in the antiretroviral therapy era: Multi-detector CT imaging findings and their correlation with intervention and patient outcomes

**DOI:** 10.4102/sajr.v30i1.3388

**Published:** 2026-05-15

**Authors:** S’yamthanda Zondi, Ntombizakhona Madlala, Balasoobramanien Pillay

**Affiliations:** 1 Department of Radiology, College of Health Sciences, University of KwaZulu-Natal, Durban, South Africa; 2 Department of Radiology, Harry Gwala Regional Hospital, Pietermaritzburg, South Africa; 3 Department of Vascular and Endovascular Surgery, College of Health Sciences, University of KwaZulu-Natal, Durban, South Africa; 4 Department of Vascular Surgery, Inkosi Albert Luthuli Central Hospital, Durban, South Africa

**Keywords:** HIV-associated large-vessel vasculopathy, multi-detector computed tomography, CT angiography, aneurysm, occlusive

## Abstract

**Background:**

HIV-associated large-vessel vasculopathy (HALVV), previously described as a distinct entity characterised by young patients with atypical aneurysmal sites and few traditional risk factors, is now recognised across a wider age range in the antiretroviral therapy (ART) era. It continues to demonstrate aneurysmal and occlusive patterns distinct from conventional atherosclerotic disease. Despite its clinical importance, systematic correlations between imaging findings, management strategies and outcomes remain limited, particularly in sub-Saharan Africa.

**Objectives:**

This study aimed to characterise the multi-detector computed tomography (MDCT) angiographic features of HALVV and assess their association with intervention and clinical outcomes.

**Method:**

A retrospective observational study was conducted at Inkosi Albert Luthuli Central Hospital (IALCH) between 2018 and 2023. HIV-positive adults with extracranial large-vessel vasculopathy undergoing MDCT angiography were included. Associations between imaging findings, intervention and outcomes were assessed using the Pearson’s chi-squared test (χ^2^) and logistic regression to identify predictors of adverse outcomes.

**Results:**

A total of 210 patients were included; 90 had aneurysmal disease, and 120 had occlusive disease. Fusiform aneurysms predominated (55.6%), and endovascular intervention was performed in 51.1% of cases. Device-related complications included type I and II endoleaks (13.3%) and stent failure (4.4%). Common iliac artery aneurysm independently predicted adverse device-related outcomes. Occlusive disease was predominantly long segment (85.8%), frequently multivessel (≥ 3 vessels in 70.8%) and associated with a high amputation rate (53.4%).

**Conclusion:**

Multi-detector CT angiography demonstrates distinct HALVV phenotypes with direct management implications. Endovascular treatment of aneurysmal HALVV shows favourable short-term outcomes but requires vigilant surveillance, particularly with iliac involvement. Occlusive HALVV is characterised by diffuse disease and poor limb salvage outcomes.

**Contribution:**

This study provides ART-era MDCT imaging–outcome correlations for HALVV in a South African tertiary setting.

## Introduction

HIV-associated large-vessel vasculopathy (HALVV) is recognised as a distinct clinical entity, with manifestations that include aneurysms, occlusive disease, dissections and arteriovenous fistulae. It typically affects younger individuals than those affected by conventional atherosclerosis and often involves multiple vascular territories or atypical arterial sites.^1,2^ The pathogenesis is multifactorial, incorporating chronic immune activation, endothelial dysfunction, direct and indirect viral effects, opportunistic infections and metabolic alterations associated with antiretroviral therapy (ART).^3,4^ Despite advances in ART and improvements in survival, HALVV remains an important contributor to morbidity and mortality among people living with HIV (PLHIV), particularly in sub-Saharan Africa, where the burden of disease is greatest.^5^

Vascular imaging plays a pivotal role in the diagnosis, treatment planning and follow-up of HALVV. Multi-detector computed tomography (MDCT) angiography provides high-resolution, three-dimensional visualisation of the vascular tree, allowing detailed assessment of vascular pathology, lesion length and extent, and calcific burden. These imaging features are crucial in guiding management decisions. Prior studies investigating HIV-associated vasculopathy have reported characteristic angiographic findings which demonstrated that HIV-related aneurysms are often multiple and saccular. Simultaneously, occlusive disease may involve long segments with poor distal run-off. These features distinguish HALVV from conventional atherosclerotic disease, which typically presents as short-segment stenosis or occlusion, especially in older individuals with established cardiovascular risk factors.^6,7,8^

Although the clinical spectrum of HALVV has been described in case series and small retrospective studies, there is limited evidence systematically correlating MDCT imaging features with management decisions and short-term outcomes.^6,7,9,10,11^ Most of these studies focus on surgical experience, with little quantitative analysis linking imaging findings to therapeutic pathways and post-procedural results. Furthermore, factors contributing to disease expression, such as ART exposure, CD4 counts, viral suppression, smoking and co-infections, remain incompletely understood with respect to imaging phenotypes and outcomes.^12,13,14^ The absence of robust data from African cohorts, where the prevalence of HIV is highest, further highlights the need for targeted research in this area. The current study was therefore designed to characterize the MDCT angiographic features of HALVV and assess their association with intervention and clinical outcomes.

## Research methods and design

A single-centre, retrospective, observational, descriptive study design was utilised. This approach allowed for the review of existing patient data to describe disease patterns, imaging characteristics and outcomes. This design was chosen due to the rarity of HIV-associated large-vessel vasculopathy and the practicality of utilising existing records to generate meaningful insights within the study timeframe.

The study was conducted at the vascular unit in Inkosi Albert Luthuli Central Hospital (IALCH), a tertiary referral centre serving KwaZulu-Natal and surrounding regions. The radiology department provides access to advanced imaging technologies, including MDCT and works in close collaboration with the vascular surgery unit.

The target population included all adult patients (aged 18 years and older) who were HIV-infected and either admitted to or evaluated at the IALCH vascular unit between January 2018 and December 2023. Eligible patients were those diagnosed with extracranial large-vessel disease and who underwent MDCT imaging for assessment and potential vascular intervention.

Hospital records showed that approximately 100 patients with aneurysmal disease and 150 with occlusive disease met the screening criteria during the study period, and therefore all eligible cases were included. The minimum required sample size for each group was confirmed using the Epi Info sample size calculator (95% confidence level, 5% margin of error), and both groups exceeded the calculated minimum thresholds. A 10% allowance for incomplete records was incorporated, and the final expected sample sizes (90 for the aneurysmal group and 120 for the occlusive group) aligned with the available dataset.

### Statistical methods

Data were analysed using Stata version 17 (StataCorp, College Station, TX, USA). Descriptive statistics were used to summarise demographic, clinical and imaging characteristics of participants. Continuous variables were reported as means with 95% confidence intervals (CI), while categorical variables were presented as frequencies and percentages. Group differences were assessed using Pearson’s chi-square (χ^2^) or Fisher’s exact test for categorical variables and independent-samples t-tests or Mann–Whitney U tests for continuous variables, as appropriate. Logistic regression analysis was performed to identify predictors of vascular interventions and outcomes, with results expressed as odds ratios (OR), 95% CI and *p*-values. Multicollinearity was evaluated using Variance Inflation Factors (VIF) and model diagnostics, including the Hosmer–Lemeshow goodness-of-fit test, conducted to ensure validity. Statistical significance was defined as a *p*-value < 0.05.

### Ethical considerations

Ethical clearance to conduct this study was obtained from the University of KwaZulu-Natal Biomedical Research Ethics Committee (No. BREC/00007665/2024). Data were anonymised and informed consent was waived for this retrospective study.

## Results

Descriptive characteristics of the study participants are summarised in Table 1. Most participants in both groups were aged 40 years and older, accounting for 74.4% in the aneurysmal group and 76.7% in the occlusive group. Males predominated in both groups.

**TABLE 1:** Descriptive characteristics of the study participants.

Patients’ characteristics	Aneurysmal group (*n* = 90)				Occlusive group (*n* = 120)			
	Frequency				Frequency			
	*n*	%	Mean	95% CI	*n*	%	Mean	95% CI
**Age (Years)**			47	44.78–49.93			49	46.90–51.40
≤ 39	23	25.56	-	-	28	23.33	-	-
≥ 40	67	74.44	-	-	92	76.67	-	-
**Gender**								
Female	29	32.2	-	-	32	26.7	-	-
Male	61	67.8	-	-	88	73.3	-	-
**Currently receiving ART**								
No	17	18.9	-	-	7	5.9	-	-
Yes	73	81.1	-	-	112	94.1	-	-
**Duration of ART**								
Newly diagnosed	17	34.0	-	-	7	13.5	-	-
≤ 5 years	18	36.0	-	-	19	36.5	-	-
≥ 6 years	15	30.0	-	-	24	50.0	-	-
**CD4 count (cells/mm)**			459	397.01–523.62			513	446.3–580.4
≥ 500	31	37.4	-	-	50	48.0	-	-
350–499	17	20.5	-	-	16	15.4	-	-
200–349	22	26.5	-	-	17	16.4	-	-
< 200	13	15.6	-	-	21	20.2	-	-
**Viral load (copies/mL)**			52 604.9	360.26–105 570.1			24 714.6	10 708.5–60 137.6
≤ 50	56	68.3	-	-	78	75.0	-	-
51–999	10	12.2	-	-	13	12.5	-	-
≥ 1000	16	19.5	-	-	13	12.5	-	-
**Smoking status**								
Current smoker	25	27.8	-	-	74	61.7	-	-
Former smoker	4	4.4	-	-	8	6.7	-	-
Never smoked	61	67.8	-	-	38	31.6	-	-
**Hypertension**								
No	57	63.3	-	-	71	59.2	-	-
Yes	33	36.7	-	-	40	40.8	-	-
**Dyslipidaemia**								
No	85	94.4	-	-	104	86.7	-	-
Yes	5	5.6	-	-	16	13.3	-	-
**TB status**								
TB positive	9	10.0	-	-	4	3.33	-	-
TB negative	81	90.0	-	-	116	96.67	-	-
**Ischaemia type**								
Acute limb ischaemia	-	-	-	-	6	5.0	-	-
Chronic limb ischaemia	-	-	-	-	95	95.0	-	-

TB, tuberculosis; ART, antiretroviral therapy; CD4, cluster of differentiation 4 (CD4+ T lymphocyte count); CI, confidence interval.

Most participants were receiving ART, although a greater proportion of aneurysmal patients were not on ART compared with the occlusive group (18.9% versus 5.9%). Newly diagnosed participants were more frequent in the aneurysmal group (34.0%), and prolonged ART exposure of 6 years or more was more common in the occlusive group.

A higher proportion of participants with CD4 counts below 350 cells/µL was observed in the aneurysmal group, while CD4 counts of 500 cells/µL or higher were more frequent in the occlusive group. Viral suppression (≤ 50 copies/mL) was common in both groups. A greater proportion of aneurysmal patients (19.5%) had a viral load ≥ 1000 copies/mL.

Current smoking was reported by 27.8% of the aneurysmal group and 61.7% of the occlusive group. Hypertension and dyslipidaemia were more frequent in the occlusive group, and tuberculosis co-infection was documented in 10.0% of aneurysmal patients (Table 1).

MDCT angiography demonstrated distinct imaging patterns between the aneurysmal and occlusive groups (Figure 2 to Figure 6). Within the aneurysmal group, the predominant aneurysm morphology was fusiform, observed in 55.6% patients (Figure 2 and Figure 3), followed by pseudoaneurysms (32.2%) and saccular aneurysms (12.2%) (Figure 4 and Figure 5). In the occlusive group, most lesions involved long segments (85.8%), with only 14.2% affecting short segments. The distribution of vessels affected also showed distinct patterns between the groups. In the aneurysmal group, most patients (86.7%) had involvement of one to two vessels, with a mean number of 1.37 vessels (95% CI: 1.21–1.54). In contrast, occlusive participants more frequently demonstrated extensive vascular involvement, with 70.8% having three or more vessels affected and a higher mean number of 4.5 vessels (95% CI: 3.97–5.03) (Figure 6). Calcification was present in both groups. Among aneurysmal cases, 37.8% showed calcification while 62.2% did not. In the occlusive group, 41.7% demonstrated calcification and 58.3% did not.

**FIGURE 1:**
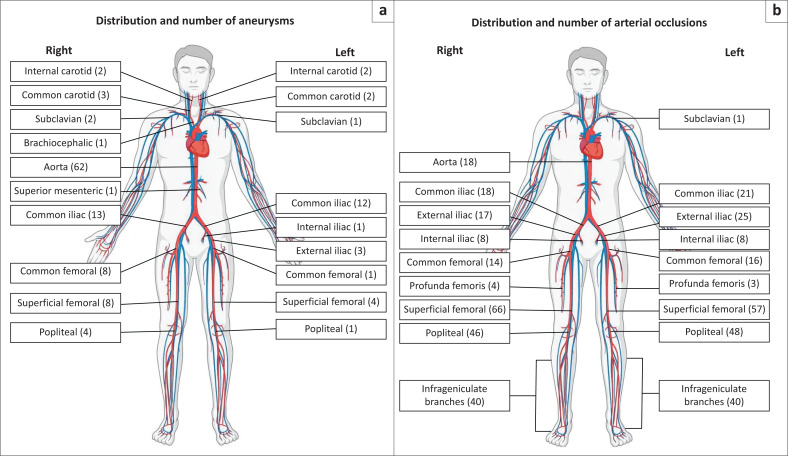
Diagrammatic representation of the anatomical distribution and frequency of vascular involvement.

**FIGURE 2:**
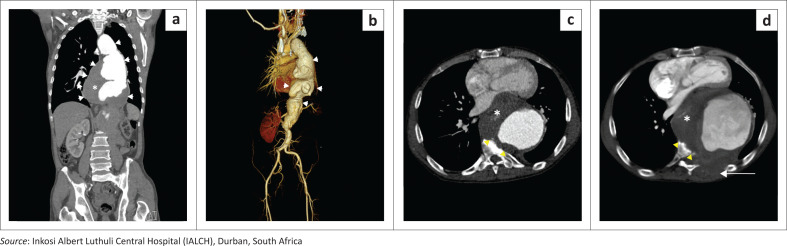
A 48-year-old man with a 3-month history of abdominal and back pain referred to the vascular unit. (a) Coronal CT angiography shows a large, multilobulated fusiform thoracoabdominal aortic aneurysm (*white arrowheads*). (b) 3D volume-rendered CT image demonstrates the fusiform aneurysm extent (*white arrowheads*). (c) Axial CTA shows anterior vertebral erosions (yellow arrowheads). (d) Follow-up CTA 1 month later shows progression of the erosions (*yellow arrowheads*) and extension of the haematoma into the posterior chest wall (*white arrow*). Eccentric intraluminal thrombus is noted (*). Surgery was not performed owing to extensive disease.

**FIGURE 3:**
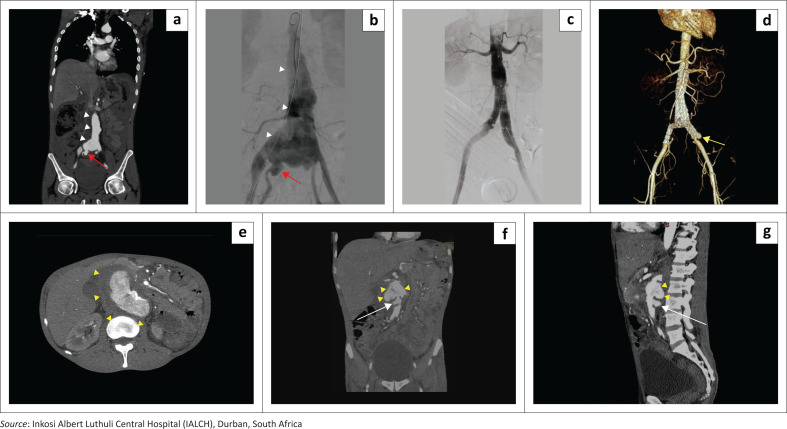
Multi-detector CT and angiographic images demonstrate variable aneurysm morphology without mural calcification in HIV-associated large-vessel vasculopathy. Panels (a–d) are from a 33-year-old man newly diagnosed with HIV who presented with acute abdominal pain and a pulsatile mass. (a) Contrast-enhanced coronal CT shows a large infrarenal fusiform abdominal aortic aneurysm (white arrowheads) with extension into the iliac arteries, and an associated saccular right internal iliac artery aneurysm (*red arrow*). (b) Digital subtraction angiography confirms the aneurysmal morphology. (c) Completion angiogram following bifurcated endovascular aneurysm repair with coiling of the aneurysmal sac demonstrates satisfactory exclusion. (d) One-year post-procedure CT angiographic 3D reconstruction shows interval development of a left common iliac artery aneurysm (yellow arrow) after defaulted follow-up. Panels (e–g) are from a different patient, a 42-year-old man, demonstrating multiple irregular, lobulated saccular aneurysms of the infrarenal abdominal aorta, with the largest (*yellow arrowheads*) revealing contained rupture. Owing to the extent of disease, surgical intervention was not performed.

**FIGURE 4:**
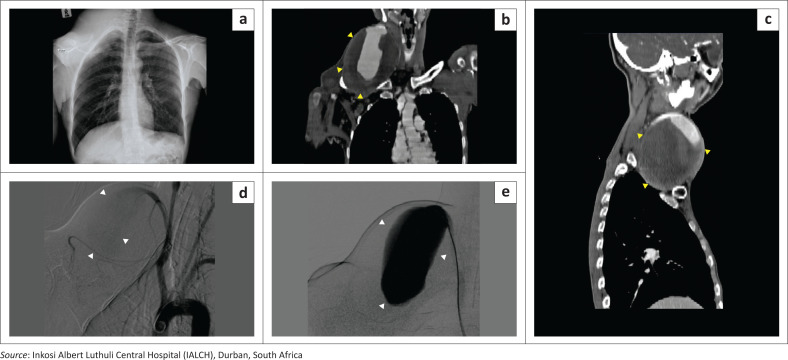
Images from a 41-year-old man with a 10-month history of a rapidly enlarging right-sided neck mass and decreased right upper-limb function. (a) Chest radiograph demonstrating a large soft-tissue mass in the right supraclavicular region without significant tracheal narrowing. (b, c) Coronal and sagittal contrast-enhanced CT angiography revealing a large saccular aneurysm arising from the right subclavian artery, measuring 10 cm, with intraluminal thrombus. (d, e) Digital subtraction angiography demonstrates opacification of the aneurysmal sac (white arrowheads). The patient declined surgical intervention.

**FIGURE 5:**
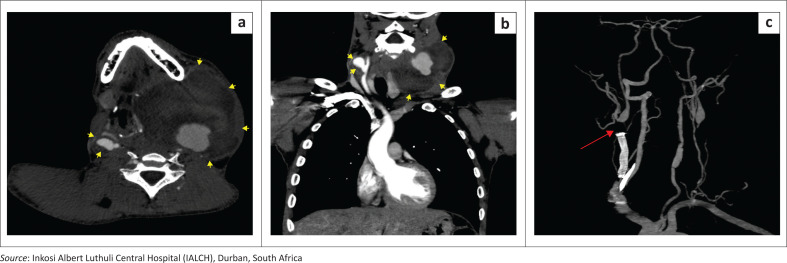
Clinical and imaging findings of a 40-year-old man on antiretroviral therapy presenting with a 3-month history of a progressively enlarging left-sided neck mass and obstructive airway symptoms. On clinical examination, a large left neck mass with overlying skin changes and blood oozing was noted. (a, b) CT demonstrated bilateral carotid aneurysmal disease (yellow arrowheads), including a ruptured left carotid pseudoaneurysm measuring 10.2 cm with a contained leak and a right carotid pseudoaneurysm measuring 2.8cm, both with circumferential intraluminal thrombi. The patient underwent open repair of the left pseudoaneurysm and right common carotid artery stenting. Re-exploration for soaked dressings revealed an intact left carotid patch with no active bleeding and evacuation of a 200-mL haematoma. (c) CTA showed occlusion of the left carotid stent and occlusion of the proximal right common carotid artery (red arrow).

**FIGURE 6:**
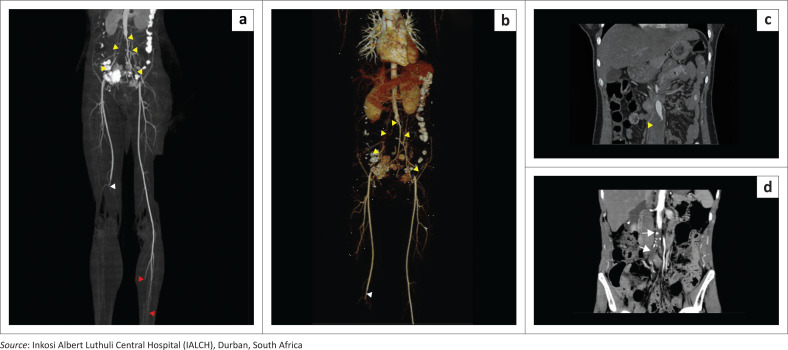
MDCT angiographic appearances of extensive occlusive disease in HIV-associated large-vessel vasculopathy across different age groups. Panels (a–c) are from a 24-year-old woman with newly diagnosed HIV who presented with acute right lower limb ischaemia. (a) 3D CT angiography demonstrated complete occlusion of the infrarenal abdominal aorta extending into both bilateral iliac arteries (yellow arrowheads), distal right SFA occlusion with absent distal opacification (white arrowhead), and left infrageniculate arterial occlusion (red arrowheads). (b) Zoomed 3D CT reconstruction confirms distal aortic and bilateral iliac occlusion. (c) Contrast-enhanced coronal CT demonstrated a non-calcified, completely occlusive thrombus in the distal abdominal aorta (yellow arrowhead). The patient underwent right leg fasciotomy, popliteal artery exploration, on-table angiography, and embolectomy, ultimately requiring right above-knee amputation for a non-viable limb. Panel (d) is from a different patient, a 49-year-old woman, and shows a completely occlusive infrarenal aortic thrombus extending into the iliac arteries with mural calcifications (white arrows) and complete bilateral popliteal artery occlusion (not displayed), representing extensive irreconstructable peripheral arterial disease. She was not a candidate for vascular intervention, and amputation was performed based on Transcutaneous partial pressure of oxygen (TcPO_2_) findings.

Open surgical repair in the aneurysmal group was performed in 13.3% of patients; endovascular intervention was more common (51.1%). Among endovascular approaches, peripheral artery stenting was used in 20.0% of patients and stent graft placement for aortic aneurysms in 31.1%. Conservative management was employed in 34.4% of patients. Amputation was rare, occurring in one patient (1.1%) who presented with a ruptured popliteal aneurysm and a non-viable limb. Post-intervention outcomes were generally positive. Endoleaks (types 1 and 2) occurred in 13.3% of patients, while stent patency was maintained in 50.0%. Stent failure occurred in 4.4% of patients and was mainly biological rather than structural or device-related. Specifically, all cases of stent failure were because of in-stent thrombosis or occlusion, not stent fracture, migration, or graft material failure (Figure 5). Additionally, re-thrombosis after initial patency was observed in 4.4% of patients. Mortality remained low within the aneurysmal group, with only three deaths (3.3%) recorded during the study period. Intervention types and outcomes in the occlusive group are displayed in Table 2.

**TABLE 2:** Distribution of intervention types and outcomes among the occlusive group.

Intervention and outcomes	*n*	%
**Angioplasty**		
No	109	92.4
Yes	9	7.6
**Embolectomy**		
No	113	95.8
Yes	5	4.2
**Stents**		
No	115	97.5
Yes	3	2.5
**Bypass**		
No	117	99.2
Yes	1	0.8
**Endarterectomy**		
No	119	99.2
Yes	1	0.8
**Primary amputations**		
No	55	46.6
Yes	63	53.4
**Best medical therapy only**		
No	74	62.7
Yes	44	37.3
**Secondary amputation**		
No	41	78.8
Yes	11	21.1
**Mortality**		
No	119	99.2
Yes	1	0.8

Figure 1 illustrates the anatomical distribution and frequency of vascular involvement identified in the study. Numeric annotations indicate the number of lesions identified at each vascular site. Infrageniculate arteries were included; despite not being classified as large-calibre vessels, as peripheral arterial involvement cannot be assessed in isolation.

### Demographic and clinical factors associated with the number of large vessels affected

In the bivariate analysis among the aneurysmal group, no statistically significant associations were observed between demographic or clinical variables and the number of large vessels affected. Factors such as age, gender, ART status and duration, CD4 count, viral load, smoking history, hypertension, dyslipidaemia and tuberculosis status did not differ significantly between patients with one to two vessels involved and those with three or more vessels affected (Table 3).

**TABLE 3:** Bivariate level association between demographic, clinical and number of large vessels affected stratified by aneurysmal and occlusive group.

Patients’ characteristics	Aneurysmal group (*n* = 90)									Occlusive group (*n* = 120)								
	1–2 large vessels (*n* = 78)					≥ 3 large vessels (*n* = 12)				1–2 large vessels (*n* = 35)					≥ 3 large vessels (*n* = 85)			
	*n*	%	Mean	95% CI	*p*-value	*n*	%	Mean	95% CI	*n*	%	Mean	95% CI	*p*-value	*n*	%	Mean	95% CI
**Age (Years)**			47.7	45.0–50.5				44.6	36.4–52.9			53.2	49.8–56.6				47.5	44.7–50.3
≤ 39	18	23.08	-	-	-	5	41.67	-	-	5	14.29	-	-	-	23	27.06	-	-
≥ 40	60	76.92	-	-	-	7	58.33	-	-	30	85.71	-	-	-	62	72.94	-	-
χ^2^	-	-	-		0.169		-	-	-	-	-	-		0.133		-	-	-
**Gender**																		
Female	23	29.5	-	-	-	6	50	-	-	11	31.4	-	-	-	21	-	-	-
Male	55	70.5	-	-	-	6	50	-	-	24	68.6	-	-	-	64	75.3	-	-
χ^2^	-	-	-		0.157		-	-	-	-	-	-		0.449		-	-	-
**Currently receiving ART**																		
No	15	19.2	-	-	-	2	16.7	-	-	1	2.9	-	-	-	6	7.1	-	-
Yes	63	80.8	-	-	-	10	83.3	-	-	33	97.1	-	-	-	79	92.9	-	-
Fischer’s exact	-	-	-		0.597		-	-	-	-	-	-		0.354		-	-	-
**Duration of ART**																		
Newly diagnosed	15	34.9	-	-	-	2	28.6	-	-	0	0.0	-	-	-	7	18.0	-	-
≤ 5 years	15	34.9	-	-	-	3	42.9	-	-	7	53.9	-	-	-	12	30.8	-	-
≥ 6 years	13	30.2	-	-	-	2	28.6	-	-	46	46.1	-	-	-	20	51.3	-	-
Fisher’s exact	-	-	-		0.913		-	-	-	-	-	-		0.179		-	-	-
**CD4 count**			452.1	383.5–520.5								554.9	443.9–666.0				497.3	413.6–581.0
≥ 500	26	36.1	-	-	-	5	45.5	-	-	19	65.5	-	-	-	31	41.3	-	-
350–499	16	22.2	-	-	-	1	9.1	-	-	3	10.3	-	-	-	13	17.3	-	-
200–349	19	26.4	-	-	-	3	27.3	-	-	3	10.3	-	-	-	14	18.7	-	-
< 200	11	15.3	-	-	-	2	18.2	-	-	4	13.8	-	-	-	17	22.7	-	-
Fisher’s exact	-	-	-		0.794		-	-	-	-	-	-		0.217		-	-	-
**Viral load**					-													
≤ 50	48	67.6	-	-	-	8	72.7	-	-	23	76.7	-	-	-	55	74.3	-	-
51–999	10	14.1	-	-	-	0	0.0	-	-	3	10.0	-	-	-	10	13.5	-	-
≥ 1000	13	18.3	-	-	-	3	27.3	-	-	4	13.3	-	-	-	9	12.2	-	-
Fisher’s exact	-	-	-		0.648		-	-	-	-	-	-		0.938		-	-	-
**Smoking status**																		
Current smoker	21	26.9	-	-	-	4	33.3	-	-	23	65.7	-	-	-	51	60.0	-	-
Former smoker	4	5.1	-	-	-	0	0.0	-	-	3	8.6	-	-	-	5	5.9	-	-
Never smoked	53	68.0	-	-	-	8	66.7	-	-	9	25.7	-	-	-	29	34.1	-	-
Fisher’s exact	-	-	-		0.854		-	-	-	-	-	-		0.581		-	-	-
**Hypertension**																		
No	52	66.7	-	-	-	5	41.7	-	-	23	65.7	-	-	-	48	56.5	-	-
Yes	26	33.3	-	-	-	7	58.3	-	-	12	34.3	-	-	-	37	43.5	-	-
Fisher’s exact	-	-	-		0.094		-	-	-	-	-	-		0.349		-	-	-
**Dyslipidaemia**																		
No	74	94.9	-	-	-	11	91.7	-	-	32	91.4	-	-	-	72	84.7	-	-
Yes	4	5.1	-	-	-	1	8.3	-	-	3	8.6	-	-	-	13	15.3	-	-
Fisher’s exact	-	-	-		0.520		-	-	-	-	-	-		0.251		-	-	-
**TB status**																		
TB positive	9	11.5	-	-	-	0	0.0	-	-	0	0.0	-	-	-	3	3.6	-	-
TB negative	69	88.5	-	-	-	12	100.0	-	-	35	100.0	-	-	-	81	96.4	-	-
Fisher’s exact	-	-	-		0.648		-	-	-	-	-	-		0.348		-	-	-
**Ischaemia type**																		
Acute limb ischaemia	-	-	-	-	-	-	-	-	-	1	2.9	-	-	-	5	5.9	-	-
Chronic limb ischaemia	-	-	-	-	-	-	-	-	-	34	97.1	-	-	-	80	94.1	-	-
Fisher’s exact	-	-	-	-	-		-	-	-	-	-	-	-	0.434		-	-	-

ART, antiretroviral therapy; CD4, cluster of differentiation 4 (CD4+ T lymphocyte count); TB, tuberculosis; CI, confidence interval.

In the occlusive group, most variables also showed no significant associations with the extent of vascular involvement. All other clinical and demographic factors, including ART use, CD4 count, viral suppression and cardiovascular risk factors, were not statistically significant with respect to the extent of occlusive involvement (Table 3).

In the aneurysmal group, none of the demographic or clinical characteristics retained statistical significance after multivariable adjustment. Although variables such as age ≥ 40 years (OR 0.42, 95% CI 0.12–1.48, *p* = 0.178), CD4 categories, ART duration and hypertension showed large effect estimates, none met the threshold for significance (Table 4). The associations seen at the bivariate level did not persist once confounding factors were accounted for.

**TABLE 4:** Multivariable level association between demographic, clinical and number of large vessels affected stratified by aneurysmal and occlusive group.

Patients’ characteristics	Aneurysmal group (*n* = 90)			Occlusive group (*n* = 120)		
	Odds ratio	*p*	95% CI	Odds ratio	*p*	95% CI
**Age (years)**						
≤ 39	1.00	-	-	-	-	-
≥ 40	0.42	0.178	0.12–1.48	4.37	**0.000**	**3.17–5.08**
**Gender**						
Female	1.00	-	-	1.00	-	-
Male	0.30	0.519	0.01–11.30	5.66	0.379	0.12–2.13
**Currently receiving ART**						
No	1.00	-	-	1.00	-	-
Yes	15.49	0.287	0.11–24.14	-	-	-
**Duration of ART**						
Newly diagnosed	1.00	-	-	1.00	-	-
≤ 5 years	10.51	0.407	0.40–27.36	-	-	-
≥ 6 years	-	-	-	-	-	-
**CD4 count**						
≥ 500	1.00	-	-	1.00	-	-
350–499	2.06	0.754	0.21–19.37	18.00	0.163	0.31–46-61
200–349	1.44	0.873	0.16–13.89	44.04	0.223	0.11–19.41
< 200	0.63	0.874	0.00–20.76	22.01	0.036	1.41–56.84
**Viral load**						
≤ 50	1.00	-	-	1.00	-	-
51–999	-	-	-	-	-	-
≥ 1000	30.61	0.462	0.00–50.42	-	-	-
**Smoking status**						
Current smoker	1.00	-	-	1.00	-	-
Former smoker	-	-	-	-	-	-
Never smoked	0.03	0.120	0.00–2.40	-	-	-
**Hypertension**						
No	1.00	-	-	1.00	-	-
Yes	14.15	0.234	11.09–18.05	-	-	-
**Dyslipidaemia**						
No	1.00	-	-	1.00	-	-
Yes	-	-	-	6.72	0.186	0.39–13.04
**TB status**						
TB positive	1.00	-	-	1.00	-	-
TB negative	-	-	-	-	-	-
**Ischaemia type**						
Acute limb ischaemia	-	-	-	1.00	-	-
Chronic limb ischaemia	-	-	-	-	-	-

TB, tuberculosis; CD4, cluster of differentiation 4 (CD4+ T lymphocyte count); ART, antiretroviral therapy; CI, confidence interval.

In contrast, in the occlusive group, older age (≥ 40 years) was strongly associated with a higher likelihood of ≥ 3 large vessels being affected (OR 4.37, 95% CI 3.17–5.08, *p* < 0.001). Furthermore, a CD4 count < 200 cells/µL was significantly associated with extensive occlusive involvement (OR 22.01, 95% CI 1.41–56.84, *p* = 0.036), suggesting advanced immunosuppression as an independent risk factor for severe disease (Table 4).

In contrast, other clinical variables, such as ART status, viral load, smoking, hypertension, dyslipidaemia and TB status, did not demonstrate independent associations once adjusted.

## Discussion

HIV remains one of the most significant health burdens globally, with an estimated 39 million people living with the virus in 2023.^15^ Sub-Saharan Africa (SSA) continues to carry the highest prevalence, with South Africa accounting for more than 7.5 million cases^16^ and in South Africa, KZN remains the epicentre of the epidemic, with adult HIV prevalence exceeding 27%, making it one of the highest-burden regions worldwide.^16^ As the HIV-positive population survives longer because of ART, long-term non-communicable complications such as vasculopathy have become increasingly recognised.^6^ Understanding HALVV in this context is essential, especially in a high-prevalence and resource-limited environment such as KwaZulu-Natal. Importantly, despite two decades of ART availability, the specific role of ART in the pathogenesis, progression and phenotype of HALVV remains incompletely understood, making this an important area of ongoing research, especially in a study focused on the ART era.

Historically, HALVV was described as a unique vascular pathology affecting young adults, characterised by aneurysms at atypical sites, a preserved atherosclerotic risk profile and evidence of arterial wall inflammation.^5,17,18,19,20^ Early reports from South Africa and other parts of Africa, mainly conducted before the widespread availability of ART, highlighted the unusual nature of the disease and its prevalence among patients younger than 40 years.^1,18,19,21^ However, these studies were small, retrospective and relied heavily on observational case series, providing limited insight into the underlying immunological or molecular mechanisms. With the advent and expansion of ART, HIV has evolved into a chronic systemic inflammatory condition, and the vascular manifestations have changed considerably. Although vascular manifestations have evolved, the independent contribution of ART, whether protective, harmful or mixed, remains unclear. This uncertainty has important implications for understanding HALVV in the ART era.

This study provides one of the most detailed single-centre MDCT-based characterisations of extracranial HALVV in the modern ART era. A key finding is the shift in age distribution; most patients in both the aneurysmal and occlusive groups are aged 40 years or older. This sharply contrasts with pre-ART descriptions and likely reflects improved longevity, chronic vascular inflammation, more prolonged ART exposure and an increasing burden of conventional cardiovascular risk factors among PLHIV.^12^ Despite this shift, HALVV continues to demonstrate demographic features described previously, including a strong male predominance, consistent with regional patterns of HIV epidemiology and health-seeking behaviour.^22^

Differences between aneurysmal and occlusive diseases were also significant. A greater proportion of aneurysmal patients were ART-naïve, while prolonged ART exposure was more common in the occlusive group. This suggests that advanced, untreated HIV and severe immunosuppression may increase the risk of arterial wall weakening and aneurysm formation. In contrast, chronic ART-related inflammation and metabolic disturbances could contribute to occlusive pathology.^5,11,23^ However, these associations do not establish causality and the precise mechanisms through which ART influences HALVV, either by modifying immune activation, promoting metabolic dysfunction or simply reflecting survival differences, remain poorly understood. Supporting this, older age and CD4 counts below 200 cells/µL independently predicted extensive occlusive involvement in this study. These findings align with international data linking immune dysregulation to endothelial dysfunction, vasculitis and prothrombotic states among PLHIV.^24^

MDCT played a crucial role in assessing HALVV, enabling clear differentiation between aneurysmal and occlusive patterns. Aneurysmal disease in this study was mainly characterised by fusiform morphology, limited multi-vessel involvement, and frequent involvement of the iliac artery. These findings are consistent with older African series and differ from Western cohorts, where aortic aneurysms and multi-territory aneurysms are more common.^3^ In contrast, occlusive disease showed diffuse, long-segment, multilevel involvement with poor distal run-off. This pattern appears more aggressive than typical atherosclerotic peripheral arterial disease and has significant implications for revascularisation feasibility and limb salvage.^5^

Management patterns revealed several important observations. A large proportion of patients with aortic aneurysms underwent open surgical repair. This is notable because many earlier studies reported poor outcomes with open surgery in HIV-positive patients.^25^ Improvements in perioperative optimisation and careful selection of suitable candidates may partly explain this shift. Endovascular therapy was preferred for iliac and peripheral aneurysms. It showed favourable short-term results, although common iliac aneurysms still had higher endoleak rates, highlighting the technical difficulty of achieving durable seal zones in arteries affected by HIV.^26^

In the occlusive disease group, revascularisation was possible in only a minority of patients because of the extensive nature of the disease, and primary amputation rates were high. These findings reflect international reports showing that HIV-associated occlusive disease often presents late, progresses quickly and carries a significant risk of limb loss, despite intervention.^27^ Logistic regression analyses further identified low CD4 count and older age as predictors of more extensive occlusive disease, emphasising the role of immune dysfunction in determining disease severity.

When viewed on a global scale, the results of this study emphasise notable differences between HALVV in high-burden African settings and the presentations described in Western literature. Western cohorts typically report older patients, greater aortic involvement and a higher prevalence of saccular morphology.^28,29,30,31^ In contrast, the current study identified significant iliac involvement in aneurysmal disease and extensive peripheral, multilevel occlusion in occlusive disease. These differences may stem from distinct genetic backgrounds, chronic inflammatory conditions, socioeconomic factors and variations in ART coverage and adherence. The contribution of ART regimen type, treatment duration and timing remains uncertain, highlighting gaps in current knowledge regarding ART-specific vascular effects.

Socioeconomic factors also shape patterns of presentation and outcomes. Many patients lack access to regular vascular screening or present late because of resource limitations, contributing to advanced disease at diagnosis. Short-term outcomes are therefore the most practical to evaluate, as long-term follow-up is restricted by mobility, healthcare access and system constraints. Despite these challenges, MDCT proved invaluable for mapping arterial anatomy, assessing suitability for intervention and guiding decision-making across both disease phenotypes.

This study supports emerging evidence that HALVV is changing in the ART era. While aneurysmal disease still displays features of the classical phenotype described in earlier African cohorts, such as presentation in relatively young patients and involvement of unusual vascular territories, there is now a clear shift. Occlusive disease appears increasingly common, more extensive and more clinically significant. These trends likely reflect the complex interactions among ongoing immune activation, ART-related metabolic disturbances and the longer lifespan of PLHIV. Collectively, these factors may be transforming the vascular landscape of HALVV, indicating a shift from the predominantly aneurysmal pattern historically observed, to a broader range of vascular pathologies. The exact role of ART, whether predominantly protective, potentially harmful through metabolic pathways or variably influential depending on regimen, remains insufficiently defined and represents a critical area for future research.

### Limitations

This study was conducted at a single centre with a relatively small sample size, which may limit the generalisability of the findings to other populations and healthcare settings. The retrospective design also relied on the accuracy and completeness of existing medical records, introducing potential information and selection bias. In addition, the absence of long-term follow-up restricted the ability to assess the durability of interventions and long-term patient outcomes. Despite these limitations, this study remains important and provides foundational evidence that paves the way for future prospective and mechanistic research on HIV-associated large-vessel vasculopathy.

### Recommendations

Future research on HALVV should extend beyond radiological and clinical characterisation to encompass mechanistic and population-level studies. Prospective multicentre research is essential to understand the natural history of HALVV in the ART era, especially how it evolves from early vascular inflammation to advanced aneurysmal or occlusive disease. Studies should also include patient, family and community perspectives, investigating barriers to early vascular presentation and adherence to follow-up. Further investigation is needed into how HIV-related immune dysregulation, ART duration and metabolic complications interact to cause vessel wall pathology. Moreover, implementation research should focus on integrating vascular imaging and surveillance into routine HIV care in resource-limited settings. Future investigations should also identify cost-effective screening methods, examine long-term outcomes following endovascular and open repair, and explore ways to incorporate HALVV management into broader national health reforms, such as the proposed National Health Insurance framework in South Africa.

## Conclusion

The study’s findings highlight the importance of imaging and HIV-related factors in the presentation of HALVV at Inkosi Albert Luthuli Central Hospital in Durban, South Africa. Traditional cardiovascular risk factors (smoking, hypertension, dyslipidaemia) did not retain independent associations with the extent of the disease after adjustment. In contrast, age ≥ 40 years and CD4 < 200 cells/µL were strongly associated with extensive occlusive involvement. Aneurysmal disease mainly displayed fusiform morphology with frequent endovascular interventions and low in-hospital mortality, whereas occlusive disease often involved widespread long-segment multivessel disease and high primary amputation rates. These patterns suggest increased vulnerability among PLHIV with advanced immunosuppression and emphasise the need for targeted, anatomy-guided care, including vigilance for iliac device-related complications.

Clinical and service interventions should prioritise comprehensive MDCT angiography mapping at presentation, integrated HIV-vascular pathways that combine urgent limb assessment with optimisation of antiretroviral therapy and immune recovery, routine screening and management of comorbid conditions, smoking cessation support and standardised post-procedural surveillance. Future research should adopt prospective, multicentre, longitudinal designs with standardised MDCT reporting and predefined outcome measures to clarify the causal relationships between immunovirological status, imaging phenotypes, therapeutic strategies and long-term limb and survival outcomes in high-burden settings.
